# Effectiveness of focal muscle vibrations in improving sensorimotor performance, mobility and strength in spinal cord injury population: a systematic review

**DOI:** 10.1136/bmjopen-2025-110054

**Published:** 2025-12-25

**Authors:** Moeez Ashfaque, Amit N Pujari, Imran Khan Niazi, Imran Amjad, Heidi Haavik, Simon F Farmer

**Affiliations:** 1 School of Physics Engineering and Computer Science, University of Hertfordshire, Hatfield, UK; 2 School of Engineering, University of Aberdeen, Aberdeen, UK; 3 New Zealand College of Chiropractic, Auckland, New Zealand; 4 Auckland University of Technology, Auckland, New Zealand; 5 Department of Health Science and Technology, Aalborg University, Aalborg, Denmark; 6 Department of Biomedical Engineering, Riphah International University, Islamabad, Pakistan; 7 New Zealand College of Chiropractic, Mount Wellington, Auckland, New Zealand; 8 University College London Hospitals NHS Foundation Trust, London, UK; 9 University College London, London, UK

**Keywords:** REHABILITATION MEDICINE, NEUROLOGY, Neurological injury, Wearable Devices

## Abstract

**Abstract:**

**Objective:**

Spinal cord injury (SCI) results in debilitating sensory, functional deficits and paralysis requiring neurorehabilitation solutions. In this regard, focal muscle vibration (FMV) is an emerging neuro-rehabilitation tool that uses mechanical vibration on muscles/tendons to stimulate underlying nerves and consequently modulate neural pathways. We conducted a systematic review to understand the exact effectiveness of FMVs on the sensorimotor function and mobility/strength in the SCI population.

**Design:**

Systematic review using the Preferred Reporting Items for Systematic Reviews and Meta-Analyses (PRISMA) approach.

**Data sources:**

PEDro, Springer, PubMed, Science Direct, Cochrane Library and Google Scholar were searched through 15 February 2025.

**Eligibility criteria for selecting studies:**

We included studies adhering to the following population–intervention–comparison–outcomes (PICO) elements. Population: SCI, intervention: FMV, comparison: unexposed controls, outcome: either of sensorimotor function or mobility and strength.

**Data extraction and synthesis:**

Two independent reviewers used standardised methods to search, screen and code included studies. Risk of bias was assessed using the Risk Of Bias In Non-randomised Studies - of Interventions (ROBINS-I) scale. Findings were summarised and a narrative synthesis is provided.

**Results:**

25 studies were included. 9 studies used FMV in the upper limb and 14 in the lower limb. The analysis includes 427 patients with SCI, with a focus on male, chronic SCI cases and a prevalence of North American studies.

**Conclusion:**

Our systematic review of 25 studies, with 21 (84%) reporting positive outcomes, suggests that FMV may improve sensory perception, motor function, mobility and strength in individuals with SCIs, with benefits observed in both limbs. However, substantial heterogeneity in FMV parameters, study designs, participant characteristics and the high prevalence of serious/critical risk of bias (13/25 studies, 52%) limit definitive conclusions. Further research with optimised protocols, larger sample sizes and longitudinal designs is needed to confirm efficacy and establish clinical guidelines.

**STRENGTHS AND LIMITATIONS OF THIS STUDY**

- This protocol follows Preferred Reporting Items for Systematic Reviews and Meta-Analyses 2020 guidance for the conduct and reporting of systematic reviews.
- The literature search includes original articles from PEDro, Springer, Science Direct, Cochrane Library, Google Scholar and PubMed in English.
- The Risk Of Bias In Non-randomised Studies - of Interventions (ROBINS-I) scale is used to evaluate the strength and quality of the evidence in the non-randomised studies.
- The scope of the review is broad, resulting in heterogeneity of the outcome.

## Introduction

 A spinal cord injury (SCI) is a damage to the spinal cord, causing paralysis and sensory deficits below the injury level. It is characterised by the disruption of sensory and motor pathways and often results in debilitating functional deficits.^1^ It can be traumatic or non-traumatic, acute or chronic, paraplegic or tetraplegic and complete or incomplete. Mobility, motor and sensory function loss and strength decrease are among the major complications associated with the SCI.^2^ Neurogenic bladder and bowel, urinary tract infections and pressure ulcers are also frequent complications.^1^ These complications negatively affect patient’s life expectancy and quality of life.^1^ The global incidence of SCIs is estimated at 105 cases per million people (19 in the UK, 40 in the US), with over 750 000 new cases projected annually^3^,^5^—80% of which are male.^6^ SCI imparts a big financial burden over the treatment bodies with costs calculated at 49.4 million US$ per decade in the US^5^ and 1.12 million GBP per patient in the UK^4^ and could even lead to poverty in low-income countries.^7^ SCI results in life-altering physical and sensory impairments, necessitating comprehensive care and rehabilitation.^1^ The treatment landscape for SCIs encompasses a range of modalities, including surgical interventions, pharmacological therapies and rehabilitative approaches.^8 9^ While surgical procedures aim to stabilise and repair the spinal cord, pharmacological treatments focus on managing symptoms like pain and spasticity. Rehabilitation plays a pivotal role in optimising function and quality of life for individuals with SCIs, encompassing physical therapy, occupational therapy and assistive devices.^18^,^10^

One emerging therapeutic modality that has received increasing interest in the past two decades is the application of vibrations, particularly focal muscle vibration (FMV), a non-invasive neuro-modulatory intervention that activates muscle fibres externally using targeted mechanical vibrations. Neurological injuries usually impart fixed changes in the organisation of the underlying neural networks, leading to disability. It is thought that FMV has the potential to tap into these neuronal networks and induce long-term depression-like plasticity in specific spinal cord circuits depending on the muscle vibrated.^11^ Consequently, a growing number of studies are exploring the role of FMV in the functional recovery in neurological injuries.^12^,^15^ FMV involves the application of mechanical vibrations to specific muscle groups or tendons. These controlled vibrations alter transmission of primary and secondary muscle afferents (Ia, Ib and type II afferents),^16^,^18^ cutaneous mechanoreceptors^19^ and modulate cortical excitability.^20 21^ Due to its ability to tap into afferent receptors and thereby modulate cortical excitability, FMV is gaining increasing interest in neurological disease management^12 14^ and is being explored as an innovative primary and adjunctive therapy in various medical fields, including SCI rehabilitation to facilitate functional recovery and improve the overall quality of life for individuals with neurological impairments.^1222^,^25^ FMV offers a distinct advantage in SCI management by providing a safe and targeted approach to neuromodulation. Unlike invasive procedures, FMV does not require surgical intervention, and it is easy to use, minimising associated risks. Pharmaceutical approaches (eg, anti-spasticity agents) are typically non-targeted and generally result in overall neural activity suppression^12^ and possible side effects.^12^ The ability of FMV to selectively target muscles and sensory receptors makes it a promising tool for enhancing muscle strength, reducing spasticity and improving sensory perception, all of which are critical aspects of SCI recovery and rehabilitation. FMV’s non-invasive nature has the potential to make it a valuable complement to the existing treatment options for spinal cord injuries.

However, despite its potential benefits, much of the research on the use of FMV has been focused on its use in stroke rehabilitation.^26 27^ As a result, the utility and effectiveness of the FMV in SCI rehabilitation remains unclear. It is cheap, safe and easy to use and suitable for lower/medium income countries. Therefore, this systematic review endeavours to explore and critically evaluate the existing body of literature ‘on the use of FMV to improve various aspects of spinal cord injury-related detriments’, particularly its effect on two critical areas of SCI recovery: (1) muscle strength and mobility and (2) sensory and motor function in patients with SCI. Both of these are important because the first lets us know about the underlying mechanisms that govern changes in the behaviour of these patients, while the second provides insights into how application of FMV translates into performance improvements. By synthesising the current evidence, this review aims to provide valuable insights into the potential efficacy and safety of FMV therapy in the management of SCI.

## Methods

### Patient and public involvement statement

As a systematic literature review, no patients and the public were involved.

This review addressed the question: how does application of FMV in SCI population help improve the mechanistic understanding of the sensorimotor function and its role to improve their functional mobility and strength? Preferred Reporting Items for Systematic Reviews and Meta-Analyses (PRISMA)^28^ reporting methods were adopted.

### Eligibility criteria

The effectiveness review was designed according to the population–intervention–comparison–outcomes (PICO) format as follows:

#### Participants

Studies that were conducted on any SCI participant, any age group or disease classification.

#### Intervention

Studies where any type of FMV was administered regardless of the intervention duration, parameters and variable.

#### Comparison

SCIs who were exposed versus people who were not exposed to FMVs.

#### Outcome

1. Studies that assessed the sensory and motor function.
2. Studies that assessed mobility and strength.

### Inclusion criteria

To be included in the review, an article had to meet the following criteria:

1. Studies that assessed the application of FMV on patients with SCI and followed the listed PICO parameters listed above.
2. Measured one or more of the outcome criteria listed above.
3. Should be conducted on humans and not in animals.
4. Published in English language.
5. Not be a thesis.
6. Should not be investigating penile vibration, or sexual or related functions.

### Information sources

The following information sources, Science Direct, PubMed, Cochrane Library, PEDro, Google Scholar and Springer databases, were used to conduct the literature search.

### Search strategy

**Figure 1:**
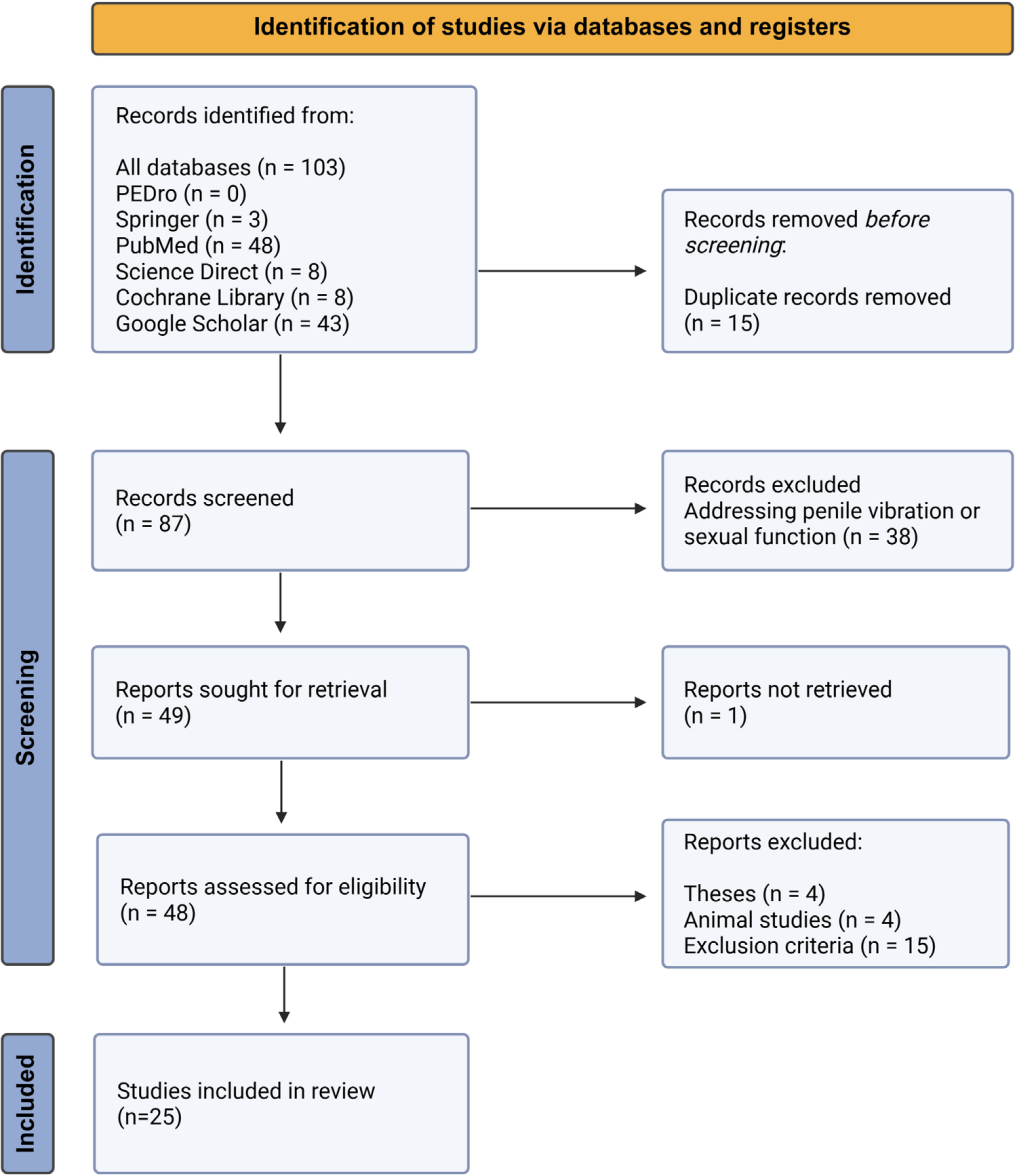
PRISMA flow diagram for paper identification and screening. PRISMA, Preferred Reporting Items for Systematic Reviews and Meta-Analyses.

The keywords used for the search are provided in box 1. Articles published until February 2025 were searched. The titles were screened initially, followed by abstracts and then full texts. Any theses/doctoral dissertations were not considered as it was not clear if they were peer reviewed. One review article was also removed for not being an original research article.^12^ Further, if articles were investigating penile vibration, or sexual or related functions, they were also screened out. Notably, many articles fell into this category and may be suitable for a separate future systematic review. Details of screening are outlined in figure 1. Due to heterogeneity of the outcomes, no meta-analysis was performed.

Box 1 **List of the keywords used**

**PEDro, Springer, Science Direct and Cochrane Library**

“spinal cord injury”, muscle vibration

**Google Scholar**

“focal muscle vibration” OR “local muscle vibration” OR “segmental muscle vibration” OR “localized mechanical vibration” OR “focal tendon vibration” OR “muscle vibration” OR “Focal Vibro-Tactile Stimulation” OR “hand-arm vibration” OR “focal vibration” OR “proprioceptive stimulation” OR “repetitive sensory input” OR “vibration” OR “vibration stimulation” OR “local vibration” OR “localized vibration” AND “spinal cord injury” OR “Tetraplegia” OR “quadriplegia” OR “paraplegia” OR “spasticity” OR “spasm” OR “spastic paraplegia” OR “spastic paresis” OR “spinal cord lesion” OR “traumatic spinal lesion” OR “hypertonia”

**PubMed**

“Spinal Cord Injuries”[(Mesh]) OR “Quadriplegia”[(Mesh]) OR “Paraplegia”[(Mesh]) OR “spinal cord lesion”[(tw]) OR “traumatic spinal cord lesion” OR “Spasm”[(Mesh]) OR “spastic*“[(tw]) OR “hyperton*“[(tw]) OR “spastic paresis”[(tw]) AND “focal muscle vibration”[(tw]) OR “local muscle vibration”[(tw]) OR “segmental muscle vibration”[(tw]) OR “mechanical vibration”[(tw]) OR “tendon vibration”[(tw]) OR “muscle vibration”[(tw]) OR “focal vibration”[(tw]) OR “vibration”[(tw]) OR “vibration stimulation”[(tw]) OR “local vibration”[(tw]) OR “localized vibration”[(tw]) OR “Vibrotactile Stimulation”[(tw]) OR “hand-arm vibration”[(tw]) OR “proprioceptive stimulation”[(tw]) OR “repetitive sensory input”[(tw]).

### Screening abstracts

Article search and screening for eligibility was performed by one reviewer (MA) that adhered to the inclusion criteria. Full text articles were obtained for all the articles that fulfilled the inclusion criteria. Two reviewers (MA and AP) assessed these articles for the screening criteria and any conflicts were mutually resolved.

### Data extraction

Data extraction was performed by the first reviewer (MA), which was then reassessed by the second reviewer (AP), and any outstanding conflicts were resolved by a mutual consensus.

The following information was gathered from each included study:

1. **Meta data:** author, year and country of the study, study design.
2. **Outcomes:** outcome measures.
3. **Participant demographics:** mean age, gender.
4. **SCI characteristics:** chronic/acute, complete/incomplete, medications, sample size.
5. **Intervention elements:** device details, vibration frequency and amplitude, duration, sessions, muscle location and type (antagonist/agonist), limb of therapy.

All data except the meta data and medication are extracted separately for intervention to the upper limb (UL) and the lower limb (LL). This is because of the disease characteristics of SCI, which can either affect the LL only (paraplegia) or all four limbs (tetraplegia). Hence, the mechanism of FMV action can be different, and a separate presentation provides an individual picture for these two cases. The meta data and medication are presented collectively. The findings are synthesised together based on the outcome measures and are presented in the discussion section. UL and LL findings are separated to present the limb-specific effects of intervention. Separating them out provides readers with limb-specific information associated with the disease, thereby guiding them towards directed and relevant information. Disease characteristics are also detailed in the narration while describing the results to present a holistic picture of the rehabilitation landscape of FMV.

### Methodological quality assessment

The Risk Of Bias In Non-randomised Studies - of Interventions (ROBINS-I) scale was used to assess the methodological quality and the risk of bias of the included studies. ROBINS-I is designed to assess the risk of bias in non-randomised intervention studies. It evaluates risks in seven domains including confounding, participant selection, classification of intervention, deviation from the intervention, missing data, outcome measurements and selection of reported results, to provide an overall risk of bias rating. Each domain is given one of four possible risk levels: low, moderate, serious and critical. If all domains are low risk, the overall risk is low; if at least one domain is moderate risk, overall risk is moderate; if at least one domain is serious risk, overall risk is serious; if at least one domain is critical risk, overall risk is critical. One reviewer (MA) performed the methodological quality assessment. The second reviewer (AP) re-checked for the quality assessment and any conflicts were mutually resolved. The results of the risk assessment of this review are listed in table 1.

**Table 1:** Methodological quality assessment of studies using the ROBINS-I scale

Included studies	Domains							
	1	2	3	4	5	6	7	Overall risk
Herszkowicz *et al*^29^	L	S*	L	L	L	L	L	S
Takakura *et al*^30^	L	S*	L	L	L	L	L	S
Ribot-Ciscar *et al*^31^	L	S*	L	L	L	L	L	S
Backus *et al*^32^	M†	S*	L	L	L	L	L	S
Gomes-Osman *et al*^33^	M†	L	L	L	M	L	L	M
Etoum *et al*^34^	L	C Case report	L	L	L	L	L	C
Fusco *et al*^35^	L	L	L	L	M	M Subjective measures	L	M
Vojinovic *et al*^36^	M†	C Case report	L	L	L	L	L	C
Tazoe *et al*^37^	M‡	L	L	L	L	L	L	M
Ashby *et al*^38^	S Medication	S*	L	L	L	L	L	S
Verrier *et al*^39^	M‡	S*	L	L	L	L	L	S
Taylor *et al*^40^	L	L	L	L	L	L	L	L
Calancie *et al*^41^	L	L	L	L	L	L	L	L
Hilgevoord *et al*^42^	L	L	L	L	L	L	L	L
Perez *et al*^43^	L	L	L	L	L	L	L	L
Butler *et al*^44^	L	S*	L	L	L	L	L	S
Cotey *et al*^45^	L	L	L	L	L	L	L	L
Murillo *et al*^46^	L	L	L	L	L	L	L	L
Field-Fote *et al*^47^	L	L	L	L	L	L	L	L
Onushku *et al*^48^	M†	L	L	L	L	L	L	M
Gomez-Sariano *et al*^49^	M†	L	L	L	L	L	L	M
Bochkezanian *et al*^22^	S‡	L	L	L	L	L	L	S
DeForest *et al*^50^	L	S*	L	L	L	L	L	S
Camerota *et al*^51^	L	C Case report	L	M	M	L	M	C
Sabalette *et al*^52^	S‡	S*	M	M	L	L	L	S

ROBINS-I domains: (1) confounding; (2) study selection; (3) intervention classification; (4) deviation from intervention; (5) missing data; (6) measurement outcomes; (7) selective reporting.

* These studies lack a proper control group.

† These studies had a functional task before intervention as a confounding factor.

‡ These studies had a stimulation given before the intervention as a confounding factor.

C, critical; L, low; M, moderate; ROBINS-I, Risk Of Bias In Non-randomised Studies - of Interventions; S, serious.

## Results

A total of 25 articles^2229^,^52^ were screened that qualified for the inclusion criteria. A total of 427 patients with SCI (mean age: 38.45, gender: 326 males, 87 females, 14 not reported) took part in the FMV studies. In one study,^39^ multiple sclerosis and transverse myelitis patients were also part of the study; results of the study on patients with SCI were only considered for this review. Remaining studies focused on SCI only. 269 patients were chronic, 27 were acute and the remaining 131 were not reported, 95 were complete and 174 were incomplete, while the remaining 154 were not reported to be either. In terms of countries, 14 studies are from North America (11 US, 4 Canada), 8 from Europe (3 Italy, 2 Spain and one each from the Netherlands, France and the UK), one from Australia and one from East Asia (from Japan, published in 1996). Out of the 25 studies, only 12 studies reported if an oral pharmaceutical medication was used or not. Of the reported people taking medications, baclofen was the most common (51 participants in total were reported administering it) followed by diazepam (eight participants, five of whom were part of a study on diazepam^39^). Two participants used oxybutynin, one participant each was reported for clonazepam and dantrolene sodium. One study also reported the use of the following medicines (one participant each): gabapentin, furosemide, 4-aminopyridine and doxazosin. 15 different consumer devices have been used to deliver FMVs. One study did not detail the device it used, and seven studies used custom developed devices. These details are listed in table 2. Details specific to the UL and LL for the results are listed in Use of FMV in the UL and Use of FMV in the LL sections.

**Table 2:** List of the vibration devices used in the studies

No	Device
1	Vibrameter^29^
2	Zeniter model TMT-18, Heiwa Electronic Industrial^31^
3	AMES technology^32^
4	CEN, USA^33^
5	Bosco system^34^
6	CroSystem nemoco^37 51^
7	Thrive no. 91^41^
8	Breul and Kjaer 4809 vibrator^42^
9	Heiwa Denshi, model TNT-18 vibrator^43^
10	Pro massager USJ-301^47^
11	Deep muscle stimulator^22^
12	Wahl jumbo vibrator^38 40^
13	Wahl vibrator model 4196 Sterling III^43^
14	Wahl powersage 4300^46^
15	Custom-made vibrators^3035 36 45 48^,^50^
16	Techo concept^52^
17	Detail not provided^39^

### Quality of the included studies

ROBINS-I tool was used to assess the methodological quality of the included studies that assessed each study across seven domains of potential risk of bias. Overall, the risk of bias varied across studies, with the majority demonstrating either low (7 studies) or moderate risk (5 studies), although several studies exhibited serious (10 studies) or critical (3 studies) methodological concerns. A predominant issue was the absence of an appropriate control group, which was identified in multiple studies^29^,^3238 39 44 50^ leading to a serious risk of bias in the study selection domain. Additionally, confounding factors were a significant concern in studies where functional tasks^32 33 36 48 49^ or prior stimulation^22 37 39 52^ were administered before the intervention. These factors introduce potential systematic differences between intervention and control conditions, which may affect the validity of FMV as the causal effect for the outcomes. Measurement bias was evident in^35^ relying on subjective outcome measures. Furthermore, missing data posed a moderate risk in three studies (Gomes-Osman *et al*,^33^ Fusco *et al*^35^ and Camerota *et al*^51^), potentially affecting the robustness of their findings. Notably, case reports (Etoum *et al*,^34^ Vojinovic *et al*^36^ and Camerota *et al*^51^) were classified as having a critical risk of bias due to their inherently limited methodological rigour and lack of comparative data. Despite these limitations, several studies^40^,^4345^ maintained a low risk of bias across all domains, demonstrating robust methodological designs with minimal threats to validity. These studies provide the most reliable evidence within the systematic review. However, the overall variability in methodological quality underscores the necessity for future research employing more rigorous study designs, including randomised controlled trials, to strengthen the evidence base for FMV interventions in neuromuscular rehabilitation.

### Use of FMV in the UL

Nine studies^29^,^37^ addressed the application of FMV on the UL in SCI patients with SCI. Six of which focused on understanding the sensory and motor function in patients in response to FMV^29^,^3337^ and six studies investigated the role of FMV in improving mobility and muscular strength in the UL.^31^,^36^ Different outcome measures studied are vibration sensation threshold,^29^ tonic vibration reflex (TVR),^31^ corticomotor excitability^33^ and cortical motor maps by transcranial magnetic stimulation (TMS)^37^ were studied for assessing the effects on sensory and motor function, while for assessing mobility and strength effects, electromyography (EMG) effects,^31^ force,^30^,^32^ torque,^32^ range of motion (ROM),^32 36^ grip and release test (GRT),^32^ 9 Hole Peg test,^33^ visuomotor tracking,^33^ modified Ashworth scale (MAS)^34 36^ and questionnaires for mobility^32 35 36^ were utilised. A total of 145 SCI participants were tested in the UL, with seven studies focusing on chronic SCIs (76 participants). In two studies (69 participants), it was not reported if the participants were chronic or acute. There was no study specifically targeting acute SCIs in the UL. Two studies were on complete SCIs (13 participants) and four studies on incomplete (33 participants). Two studies had both complete (8) and incomplete (27) participants, while one study did not mention the completeness details of the SCI (64 participants).

In terms of vibrational frequency for FMV, most of the studies fell within the 60–80 Hz range. Two studies used 60 Hz, one each for 66, 70 and 75 Hz, two for 80 Hz and one each for 100 Hz and 120 Hz. Only four studies reported the vibration amplitudes: two studies 2 mm, and one study each for 1 mm and 0.4 mm. Vibration was applied for less than half a minute in four studies (9 s, 15 s, 15 s and 25 s), 5 min in one study and 10 min in two studies. Two studies did not report the duration of application. Five studies had only a single session of vibration, and one each having 3, 6, 10 and 25 sessions. The following muscles were used for delivering FMVs: one study each for clavicle (skin of superior surface of the clavicle 8–10 cm from its sternal end), middle finger, wrist extensor, metacarpophalange (MCP) and first dorsal interosseous (FDI). One study applied to biceps brachii, one to triceps brachii and one applied on both biceps brachii and triceps brachii. One for flexor carpi radialis (FCR) while one for both extensor and flexor of the forearm. Of these, in five studies, FMV was applied to tendons whereas in four studies, it was applied to muscles. In four studies, the muscle group was an agonist muscle; in one, it was the antagonist, and in the other three studies, both the antagonist and agonist muscles. Please refer to the accompanying online supplemental information for the details of the UL studies.

### Use of FMV in the LL

16 studies addressed the application of FMV on the LL.^2238^,^51^ 11 studies^38^,^4446 49^ investigated the sensory and motor function, while seven^2245^,^48 51 52^ studied the effect of FMV on mobility and strength in the LL. Outcome measures used to assess the function of the participants were Hoffman reflex (H-reflex),^38^,^4449^ TVR,^38^ tendon reflex,^46^ numerical rating scale (NRS) for pain^51 52^ and EMG^4449^,^51^ for sensory and motor function, while EMG,^45 48^ MAS,^46 51^ ROM,^46 51^ trunk control test,^51^ functional ambulation categories (FAC),^51^ medical research council scale for muscle strength^51^ and torque^22 48^ were used for measuring mobility and strength. A total of 282 SCI participants were tested in the LL, with ten studies focusing only on chronic SCIs (141 participants) and in two studies (62 participants) it was unknown if the participants were chronic or acute. Four studies had both chronic and acute participants (79 individuals). There was no study specifically targeting acute SCIs in the LL. Four studies had both acute (27) and chronic (52) participants. Two studies were on complete SCIs (13 participants) and three studies on incomplete (51 participants). Eight studies had both complete (66) and incomplete (65 participants), while three studies did not mention the completeness of SCI (91 participants).

As far as vibrational frequency for FMV is concerned, 60 and 80 Hz were the most used. Five studies used 60 Hz and four used 80 Hz. Two studies of 100 Hz. One study each delivered 50 Hz, 55 Hz and 110 Hz. One study tested four different vibration frequencies: 20, 40, 80 and 120 Hz. Nine studies reported the vibration amplitudes: two 1 mm, and one each for 0.5 mm, 1.5 mm, 2.2 mm, 3 mm, 4 mm and 7 mm. One study reported 0.2–0.5 mm. One study did not report either the frequency or the amplitude of vibration.^52^ In terms of duration of vibration, vibration was applied for the duration of obtaining the H-reflex in four studies,^39^,^42^ for the duration until a TVR was obtained in one^38^ and for the duration of applying neuromuscular electrical stimulation (NMES) in one.^22^ Vibration duration was less than 20 s in four studies: 1 s in ^50^, 2 s in ^44^, 10 s in ^48^ and 15 s in ^49^. 60 s of vibrations were applied in ^45^, 3 min in ^43^, 5 min and 30 s in ^47^ and in ^46^ and^52^ vibrations were applied for 10 min. It was 80 min in ^51^. All the LL studies were a single session study, except ^51^ which had 30 sessions and ^52^ which had four sessions. Pertaining to the muscle of FMV application, six studies used the Achilles tendon, two studies delivered on tibialis anterior (TA) tendon and one study applied on both Achilles and TA tendon. Two studies used rectus femoris (RF) muscle, one study each for patellar tendon and plantar surface of foot, in one study quadriceps, hamstring and tensor fasciae latae (TFL) muscles were used, in one study quadriceps, hamstrings, gastrocnemius (GM) and iliopsoas muscles were used, and in one study knee, ankle and hip extensor and flexors were used. Most of the studies were focusing on delivering FMV to tendons (10 studies), whereas in some (6 studies) it was applied to muscles. In seven studies, the muscle group was an antagonist muscle; in five studies, it was the agonist, and in the other four studies, both the antagonist and agonist muscles were used. The accompanying online supplemental information file has details of the LL studies.

## Discussion

The results presented in this study provide valuable insights into the use of FMV as a potential therapeutic approach for individuals with SCI. The discussion will address key findings, implications and limitations of the reviewed studies. It will also shed light on the current state of knowledge on the use of FMV for improving sensory and motor function and mobility and strength in the ULs and LLs in the SCI population.

The included studies involved a total of 427 SCI patientswith SCI. 76.4% of the SCI patients were male, reflecting the higher prevalence of research participation in SCI males. This is consistent with previous observations of higher occurrence rate (about 80%) in males.^6^ Chronic SCI cases were more prevalent in the studies, indicating a focus on individuals with a later phase of the injury. Interestingly, most of the studies did not specify the completeness of the SCI, which could significantly impact the interpretation of the results. Regarding the geographical distribution of the research, the majority of studies were conducted in North America, which may be indicative of availability of resources and/or regional differences in research interest and priorities. It is important to note that there was a significant focus on chronic SCI cases in both UL and LL studies, while the representation of acute SCI cases was limited. This imbalance in participant demographics could affect the generalisability of the findings to acute SCI populations. Future research should aim to include a more diverse range of SCI types and durations to better understand the potential benefits of FMV across different phases of the injury. The studies reviewed primarily utilised vibration frequencies in the range of 60–80 Hz, with 60 Hz and 80 Hz being the most common choices. This consistency in frequency selection may indicate an established optimal range for FMV interventions. However, the vibration amplitudes and durations varied across studies, which could further influence the effectiveness of FMV. As underlying neurophysiological mechanisms determining the effects of FMV in SCI are still poorly understood, with only a handful of studies providing this mechanistic evidence,^43 47 49 50^ further research is needed to determine the ideal parameters for FMV application in SCI rehabilitation.

### Mobility and strength

#### Upper limb

As should be clear from the discussion below, the use of FMV in ULs of SCIs presents a mixed picture of its potential benefits on mobility. FMV applied to the biceps tendon in both complete and incomplete SCIs (level C4-T1) resulted in an illusion of arm movement, although smaller than in healthy individuals,^35^ suggesting potential for sensory perception in SCIs, though the extent is unclear. In contrast, a single 10 min FMV session at the FCR tendon area during a functional task had no immediate impact on mobility measures; incomplete SCI, level C4-C7.^33^ The effectiveness of FMV may depend on session duration, frequency and stimulation location. Case studies revealed promise in long-term effects. Six FMV sessions (duration: 15 min each) in forearm muscles alongside functional tasks improved the MAS and ROM; incomplete SCI, level C2-T2.^36^ Even shorter (duration: 5 min each), 10-session FMV interventions in the triceps brachii showed lasting benefits (up to a month) in the MAS; incomplete SCI, level C5.^34^ In a study involving 25 sessions of FMV in the hand region during functional tasks for 10 incomplete patients with SCI (level C2-C7), significant improvements were observed in grip and release performance as measured by the GRT and ROM.^32^ GRT showed a 23% improvement immediately after the sessions, with a 7% further improvement 3 months post-treatment. In summary, FMV’s efficacy in SCIs is context-dependent—depending on duration and length of intervention, and location of application, with potential for improvement in sensory perception and long-term benefits. The specific factors influencing its effectiveness, such as application location (tendon vs muscle), session duration and combination with functional tasks require further exploration for optimal rehabilitation in SCIs.

FMV shows promise in improving UL strength in individuals with spinal cord injuries. However, its effects vary depending on factors such as muscle function and exposure duration. In complete SCIs who had injury sustained at C2-C7 level, 9 s of FMV improved elbow extension force generation in the biceps brachii but was inconsistent in the triceps brachii, highlighting the differences in its effects on antagonist and agonist muscles.^31^ Long-term effects were not studied. A 10 min session of FMV at the FCR tendon enhanced pinch force but only temporarily; the improvements did not last 30 min post the session; incomplete SCI, level C4-C7.^33^ And 25 sessions of FMV for 30 min to the antagonist hand muscles (metacarpophalangeal joint and wrist) in incomplete SCIs (level C2-C7) while performing a functional task enhanced the muscle strength.^32^ Long-term effects were again not studied. These findings suggest FMV’s potential in enhancing UL strength, but further research is needed to understand the impact of vibration location (tendon vs muscle, agonist vs antagonist), long-term effects and differences in its effects on complete and incomplete pathologies.

#### Lower limb

FMV presents opportunities for improving LL muscle activity and gait dynamics in spinal cord injuries. However, nuances and unexplored aspects deserve attention. In one study,^45^ 54 s of FMV to the RF muscle boosted muscle activity in proximal quadriceps muscles during assisted gait but had no impact on distal leg muscles, suggesting the effects of FMV may be limited to stimulated muscle group(s) and its synergists/antagonists. In the same study, FMV also improved the transition between swing and stance phases of gait in patients with SCI (both complete and incomplete; levels C4-6, 8, T2-4) who had pathological gait. Another study^48^ demonstrated that 10 s of Achilles tendon vibration during assisted hip movement increased muscle activity in the TA and GM muscles, particularly during hip flexion and with voluntary assistance; complete SCI, level C3-C7, T5, 6. The effects of FMV with and without movement assistance, clinical outcomes and long-term implications were, however, not studied in both these studies. A different study^46^ extended FMV to 10 min on quadriceps, enhancing ROM and MAS scores in SCIs (both complete and incomplete; levels C3-7, T4, 6, 8, 9, 11), but it didn't assess muscle activity and had varied outcome measures.

Surprisingly, 5.5 min of FMV to thigh muscles induced a step-like response in chronic complete and incomplete SCIs (levels C4-7, T4, 6, 8, 9, 11),^47^ independent of SCI completeness and unaffected by locomotor training. The TFL muscle showed the most robust response. Furthermore, a study explored the combination of FMV and NMES,^22^ revealing improved muscular work capability in some SCIs, while others experienced decreased capability. These were a mix of both complete and incomplete patients, at various levels: C6, 7 T3, 5–7, 12, L3. The underlying mechanisms here remain unclear. A longer dose of FMV – 30 sessions, 80 min each—in a chronic case study (level and severity unclear) reported promising results, with increases in MAS (by two points), TRC (from 66 to 100) and FAC (from 3/5 to 4/5).^51^ It also reported improvements in stride time and walking speed. In conclusion, FMV holds promise for enhancing mobility in SCIs, but location specificity, movement assistance, clinical outcomes and long-term effects need further investigation to optimise its use in rehabilitation.

### Sensorimotor function

#### Upper limb

The presented findings provide valuable insights into sensory perception and motor function in the ULs of SCIs. Notably, individuals with SCIs at C2 and below (lower range not mentioned) have a reduced sensory threshold at the clavicle, directly correlating with the injury level.^29^ The level of completeness though was not mentioned. Furthermore, FMV interventions appear promising in restoring sensory perception, as demonstrated by significant improvements in finger digit sensation following a (30 min each) 25-session intervention; incomplete SCIs (levels C2-7).^32^ 25 s of FMV to the middle finger resulted in a finger flexion reflex in complete SCIs (levels C5, 6), the amplitude of which was inhibited via acupuncture techniques.^30^

Nine seconds of FMV always induces a TVR in the biceps brachii but only half the time in triceps brachii muscle; complete SCIs (levels C4-7).^31^ TVR is a sustained contraction of a muscle after being subjected to vibration. Vibration excites muscle spindles, which in turn induce reflex contractions in the muscle being vibrated.^31^ The inconsistent TVR response observed between the agonist and antagonist in upper arm muscles warrants investigation into its neurophysiological implications.^31^ Additionally, the impact of vibration on motor function and neuroplasticity is evident, as 10 min of FCR tendon vibration increased long-term (30 min after intervention) corticomotor excitability; incomplete SCIs, level C4-C7.^33^ This corticomotor excitability was assessed with TMS and motor evoked potentials from the thenar muscle. A similar effect was observed with FMV at the FDI tendon, where motor maps generated by TMS expanded; both complete and incomplete SCIs, level C2-5, 7, 8.^37^ The immediate corticomotor excitability in^33^ however, did not change.

#### Lower limb

In the LL, there is a larger number of studies investigating sensory and motor performance. The soleus H-reflex response to Achilles tendon vibration in the LL has been widely studied in literature and has offered valuable insights into neural mechanisms in the context of SCIs. The H-reflex is a monosynaptic reflex elicited by electrically stimulating sensory nerve fibres (Ia afferents), used to assess spinal motor neuron excitability and reflex pathways. Early studies by Ashby *et al*^38^ and subsequent validations^41 42^ have shown that, in the acute phase of SCI for both complete and incomplete cases, vibration completely diminishes the soleus H-reflex, despite higher H-reflex amplitudes without vibration compared with healthy population. The level of SCIs is though not provided. In the chronic phase, the H-reflex response ratio between vibration and no vibration (H_vib_/H) increases compared with healthy population, indicating reduced H-reflex inhibition in response to vibration.^38 41 42^

Additionally, the TVR for the soleus muscle elicited by Achilles tendon vibration is completely or almost completely diminished in acute SCI (both complete and incomplete, levels unknown), and this reduction persists in the chronic phase.^38^ The H/M ratio is the ratio of the maximal H-reflex amplitude to the maximal M-wave amplitude, reflecting spinal motor neuron excitability. A higher ratio indicates increased excitability, while a lower ratio suggests reduced excitability or inhibition. Diazepam acts on GABA-A (gamma-aminobutyric acid type A receptor) and is used in SCI to treat spasms and spasticity. Hence, diazepam’s influence on FMV action is also studied; the H_vib_/H ratio in complete SCIs (levels unknown) does not change using diazepam.^39^ However, the effect of diazepam on the H/M ratio is not known, which can provide further insights into the neural activity. Notably, as spasticity increases in patients with SCI (unknown completeness and levels), the H_vib_/H ratio also rises,^40^ signifying reduced depression of the H reflex with vibration. The ratio increased with the duration of lesion for all patients and was unrelated to the level of lesion or the completeness of SCI.^40^ Following neurophysiological implications are suggested by the author: (a) mechanisms blocking the H-reflex become less effective as spasticity gains prominence and (b) vibration produces greater background facilitation of motoneurons in spasticity conditions.^40^

Calancie^41^ noted that presynaptic inhibition is enhanced in complete and incomplete acute SCI (levels C1-7, T6, 10), contributing to hyporeflexia during spinal shock, while it is diminished in chronic SCI, contributing to hyperreflexia associated with spasticity. These studies laid the foundation for investigating complete H recruitment curves to better understand neurophysiological behaviour. Hilgervood^42^ examined M-wave characteristics and H-reflex thresholds with and without vibration, finding that vibration lowered H-reflex thresholds but did not affect the maximum H-reflex thresholds, and the M-wave remained consistent between subjects and conditions (the completeness and levels were unknown). Reduction in H-reflex due to vibration is attributed to presynaptic inhibition and post-activation depression.

Murilo^46^ extended vibration research to other muscle groups and with longer exposure time, applying 10 min of FMV to the quadriceps. This led to a decrease in the Soleus H/M ratio, with more prominent effects in complete SCIs. Vibration also reduced clonus frequency and duration and decreased the tendon (T) reflex—a monosynaptic reflex triggered by mechanically tapping a tendon, stretching the muscle and activating muscle spindles. The heteronymous H-reflex response in complete and incomplete SCIs was attributed to mechanical spread of vibration and the 'busy line’ phenomenon, making fibres unresponsive to other inputs during vibration. Perez^43^ examined the effects of focal vibration on the TA tendon in chronic complete SCIs (levels C4-7, T1, 2, 5, 8), highlighting the H-reflex behaviour in reciprocal Ia and presynaptic D1 inhibition mechanisms in the Soleus muscle. Vibration inhibited H-reflex for reciprocal Ia inhibition (maintained up to 5 min) but did not have significant short or long-term effects on presynaptic D1 inhibition. The presynaptic Ia terminal and Ia interneuron were considered the possible sites of inhibition and presynaptic inhibition; post-activation depression and robust spindle activation were attributed as the possible causes. Spasms are a common consequence after SCI. Butler^44^ found that involuntary spasm-like EMG activity, evoked by superficial nerve stimulation in complete chronic SCIs (levels C4-7, T6), was reduced by vibrating Achilles tendon. The EMG activity depression was prominent in muscles proximal to the site of vibration but not in the distant ones. The vibration, however, did not affect the SOL H-reflex in SCIs. Persistent inward currents (PICs) were attributed to the long-lasting depression of the involuntary activity and not presynaptic terminal inhibition or motoneuron excitability. Cutaneous reflex responses were also explored.

Gomez-Sariano^49^ showed that vibration inhibited the long latency TA cutaneous reflex during plantarflexions in incomplete SCIs (levels not available) with spasticity, with the extent of inhibition correlated to the MAS, indicating greater reflex inhibition in subjects with higher spasticity. In contrast, DeForest^50^ noted that tendon vibration at specific frequencies for complete and incomplete SCIs (levels C2, 4–7, T4-7, 9, 11) inhibited the long-lasting component of the cutaneous reflex in antagonist muscles but not in agonist muscles, offering insights into the suppression of PICs and the activation of interneurons involved in central pattern generator networks. More recently, Camerota^51^ showed that the pain threshold measured through NRS was increased by 30 sessions of 80 min each FMVs to a case of chronic SCI (level, severity unclear). It also reported improvements in EMG activation patterns and co-activations but failed to provide its empirical evidence. 4 sessions of 10 min of FMV combined with virtual reality also showed a decrease in NRS pain thresholds in chronic SCIs (levels C6, 7; both severities).^52^

In conclusion, analysis of the 25 included studies shows that 21 out of the 25 studies report positive outcomes in improving sensorimotor function and mobility and strength. Four studies, however, show no change or mixed results, with one study^33^ in the UL and three^22 39 44^ in the LL. However, substantial heterogeneity in FMV parameters, study designs, participant characteristics and the high prevalence of serious/critical risk of bias (13/25 studies, 52%) limits definitive conclusions. These studies contribute significantly to our understanding of neural mechanisms affected by FMV in SCIs. They highlight the potential for vibration to induce plastic changes in neural circuitry and improve spasticity management. Future research should continue to explore the implications of different vibration frequencies, stimulation location (muscle vs tendon, agonist vs antagonist), with and without accompanying muscle contraction/activity and the potential for long-term neural modulation.

## Future recommendations

The reviewed studies have provided an extensive overview of the use of FMV in SCI rehabilitation, yet several limitations exist that hinder the ability to draw robust conclusions. This section provides key considerations and recommendations for future studies to improve experimental design and enhance the field’s understanding of FMV’s effectiveness. These considerations/ recommendations are provided separately for methodological and for mechanistic improvements in the sections below.

### Methodological considerations and recommendations

#### Sample size and study design

The majority of reviewed studies included small sample sizes, often with fewer than 30 participants, which limits statistical power and generalisability. Many studies also lacked proper control groups, resulting in a high risk of bias. Future studies should prioritise randomised controlled trials with adequately powered sample sizes to increase reliability. A crossover design, where participants receive both FMV and sham interventions, could provide greater control over inter-subject variability and improve internal validity.

#### Participant diversity and stratification

A significant proportion of the studies focused on chronic SCI cases, with limited exploration of FMV’s effects in acute and subacute populations. Future research should include a broader spectrum of SCI severity (complete vs incomplete) and injury duration to understand FMV’s effects across different stages of recovery.

#### Standardisation of FMV parameters

A major limitation of existing research is the variability in FMV parameters, including frequency, amplitude, duration and the number of sessions. While 60–80 Hz appears to be the most used frequency, the optimal parameters for different outcome measures remain unclear. Future studies should systematically investigate a range of frequencies (eg, 40–120 Hz) and vibration amplitudes (eg, 0.5–4 mm) to identify the most effective combinations. Additionally, intervention duration and total number of sessions should be optimised to balance efficacy with feasibility in clinical settings.

#### Target muscle selection and stimulation site

The reviewed studies applied FMV to both muscles and tendons, yet little attention was given to the comparative efficacy of these approaches. Further research should explore whether targeting agonist versus antagonist muscles or specific muscle groups (eg, proximal vs distal muscles) leads to differential outcomes. Additionally, given that FMV’s effects may vary depending on the level of injury, future studies should tailor FMV application based on SCI level and functional impairments.

#### Outcome measures and longitudinal assessments

Many studies employed heterogeneous outcome measures, making direct comparisons challenging. Standardisation for assessment should be developed. Furthermore, it is seen in a case study^51^ that long exposure shows improvements in outcome measures; this can be extended to larger populations. Longitudinal assessments should also be prioritised to find the lasting effects of the treatment therapy.

### Mechanistic considerations

The precise mechanisms through which FMV exerts its effects on SCI populations remain poorly understood. Addressing these gaps will require mechanistic studies that elucidate the neural and physiological processes underlying FMV-induced improvements in sensorimotor function.

#### Exploration of cortical and spinal circuits

As it is seen that FMV has potential for corticomotor excitability, future studies should employ detailed cortical imaging techniques like EEG to assess cortical plasticity changes following FMV. Source localisation of EEG can provide an in-depth picture of functional changes in the brain following FMV. Alongside this, assessments of spinal excitability using H-reflex and motor evoked potentials can be helpful in getting an even more detailed picture. The effect of location (agonist/antagonist, muscle/tendon) of FMV on the H-reflex also needs to be addressed. Cortico-muscular coherence is also an area that can be explored.

#### FMV as adjunct therapy

Preliminary findings suggest that combining FMV with functional training or NMES may enhance outcomes. Future research should systematically evaluate FMV as an adjunct therapy.

#### Longitudinal studies

To assess whether FMV leads to lasting neuroplastic changes, longitudinal studies should be conducted.

## Study strength and limitation

### Study strengths

The major strengths of this review are as follows. By categorising studies based on the extracted data, our analysis provides a structured understanding of FMV’s effects on mobility, strength and sensorimotor function. It also helps in the identification of various FMV devices and parameters used in SCI research, and the effect of the use of various adjunct therapies (TMS, nerve stimulation, virtual reality) used in conjunction with FMV. This review underscores the potential of FMV as a non-invasive neuromodulatory approach, consolidating evidence on its ability to modulate sensorimotor pathways. By integrating findings across diverse experimental designs, our synthesis contributes to bridging the gap between preclinical mechanisms and clinical applications of FMV. This will aid in developing standardised protocols that can be effectively translated into rehabilitation. Additionally, this review highlights critical gaps in current research, offering specific recommendations that may guide future studies towards optimising FMV interventions in SCI populations.

### Study limitations

This systematic review has certain limitations to consider. The included studies exhibited substantial heterogeneity in design, FMV parameters and participant characteristics, making direct comparisons and generalisability challenging. Small sample sizes, limited diversity in SCI severity and duration and the absence of control groups in some studies raise questions about the robustness of the findings. Variation in vibration devices is another question. Regional bias and potential publication bias may affect the generalisability of results. Many studies focused on short-term outcomes, while long-term effects and underlying mechanisms were often underexplored. These limitations emphasise the need for more standardised, diverse and mechanistic research to better understand the potential and practical application of FMV in SCI rehabilitation.

## Conclusion

This systematic review synthesises the current evidence on the use of FMV for improving sensorimotor performance, mobility and strength in individuals with SCI. The findings from the 25 included studies suggest that FMV holds promise as a non-invasive neuromodulatory intervention. 427 SCI individuals, predominantly chronic male cases, were part of these studies, with the majority of studies in North America. A substantial majority of studies (84%) reported positive outcomes, indicating potential benefits in enhancing sensory perception, corticomotor excitability, muscle strength, ROM and spasticity management in both the ULs and LLs. The ability of FMV to modulate spinal reflex pathways, such as the H-reflex, and induce cortical changes points toward its capacity to engage neuroplastic mechanisms, offering a rationale for its therapeutic application.

However, these encouraging findings must be interpreted with considerable caution due to substantial limitations inherent in the existing literature. The high degree of heterogeneity in FMV parameters (frequency, amplitude, duration and application site), study designs, small cohorts and participant characteristics precludes the formulation of definitive conclusions or clinical recommendations. Performance variation between FMV devices^53^ can also contribute to variation in their effects. Crucially, the methodological quality of the evidence is a significant concern, with over half of the studies exhibiting a serious or critical risk of bias, often due to the absence of control groups, small sample sizes and the prevalence of case reports and non-randomised designs.

Therefore, while FMV emerges as a safe and potentially effective tool, the current evidence is insufficient to confirm its efficacy or establish standardised clinical protocols. The promising results highlighted in this review should serve as a catalyst for more rigorous, high-quality research. Future studies should prioritise randomised controlled trials with larger, more diverse cohorts, standardised outcome measures and optimised, consistent FMV parameters. Longitudinal investigations are essential to determine the persistence of benefits. Until such evidence is available, the application of FMV in clinical practice for SCI rehabilitation remains experimental, and its potential, though significant, is not yet fully substantiated.

## Supplementary material

10.1136/bmjopen-2025-110054 online supplemental file 1

## Data Availability

All data relevant to the study are included in the article or uploaded as supplementary information.
