# Supplementary material for: Effectiveness of focal muscle vibrations in improving sensorimotor performance, mobility and strength in spinal cord injury population: a systematic review
Source: BMJ Open. 2025 Dec 25;15(12):e110054. doi: 10.1136/bmjopen-2025-110054 (PMC12742131; doi:10.1136/bmjopen-2025-110054)
Supplement: Supplementary file 2 [file bmjopen-2025-110054.reviewer_comments.pdf]

PEER REVIEW HISTORY

BMJ Open publishes all reviews undertaken for accepted manuscripts. Reviewers are asked to complete a checklist review form and are provided with free text boxes to elaborate on their assessment. These free text comments are reproduced below.

ARTICLE DETAILS

Title (Provisional)

Effectiveness of Focal Muscle Vibrations in Improving Sensorimotor Performance, Mobility and Strength in Spinal Cord Injury Population: A Systematic Review

Authors

Ashfaque, Moeez; Pujari, Amit; Niazi, Imran Khan; Amjad, Imran; Haavik, H; Farmer, Simon F.

VERSION 1 - REVIEW

**Reviewer**	**1**
**Name**	**Santosh Chandrasekaran**
**Affiliation**	**Northwell Health**
**Date**	**07-Feb-2025**
**COI**	**None**

This manuscript attempts to present a systematic review on the effects of Focal Muscle Vibrations (FMVs) on sensorimotor function, and functional gains such as mobility and strength in people with SCI. They included 23 studies, 9 focusing on the upper-limb and 14 on lower-limb interventions. The studies included 422 individuals with SCI and 132 able-bodied individuals.

I have significant concerns that this review does not add to the body of the knowledge. I believe a systematic review should aim to further the field by suggesting future experiments by highlighting the gaps in the existing body of knowledge.

My detailed comments are as follows:

Abstract
- Line 35 - "The pathophysiology of SCI…less effective than desired." - This is too vague. For better readability, it should perhaps focus on what is missing and how FMV might fill that gap. Please also briefly include what FMV is and how it is administered.
- "… FMVs, through afferent nerve stimulation…" - This statement is too vague and too

strong. Please clarify.
Conclusion - "Our findings suggest potential benefits…" - On what percentage of studies and in what context is this statement based? This is again too broad and too strong a statement. Please include in the abstract, briefly, appropriate caveats and percentage of studies that support this conclusion.

Intro
• Pg 3 - This section of the Intro is too broad and decribing the statistics in a lot of detail. In my opinion, it is too distracting and I would encourage the authors to make it more concise for better readability. Please restructure in a way that it flows for the introduction of FMV in the next section.
• Line 40 - "and, pressure ulcers"
• Line 41 - complications negatively affect patient's life expectancy
• Pg 4, line 6 - "
inherent potential of neuromuscular system for
promoting functional recovery after neurological injury (such as SCI) or neurological disorders" This is extremely unclear and vague. Please clarify. What do the authors mean by "The neuromuscular system having an inherent potential to promote recovery"? Please introduce and explain this in multiple sentences.

Methods

Results
• Stage and LC needs to be rephrased for better readability

Discussion
Section 4.1
• Please have separate headings/sections for UL and LL
• Line 27: "mixed picture of its potential benefits..". This language conflicts with the one in the Abstract - "Our findings suggest potential benefits…". Please edit.
• Line 37: mention level of completeness of SCI
• Line 41: context-dependent. Please be explicit
• Line 49: C7 or higher - how high? That might explain why triceps did not show improvement
• Pg 14, Line 4 onwards: The authors keep emphasizing that SCI context matters to evluaate FMV effectiveness but seldom mention the context when describing the observed results. I would encourage the authors to mention level of SCI and completeness
• Line 17: "brief 5.5 minutes" how is it brief when there's a mention of a 54s FMV intervention
Section 4.2
• Pg 14, line 36: TVR abbreviation

• Line 44: how was corticomotor excitability evaluated?
• Line 60: Please mention what is the clinical effect of Diazepam to provide context - GABA agonist
• Line 60: Please define H/M ratio
• Please define T-reflex

The authors went to great lengths to list out all the variables encountered in the studies they review - outcome measures, vibrational frequency, vibration amplitudes, duration of FMV administration, number of sessions, relation between target muscle and injury level. However, they make no recommendations for future studies based on these observations. I would encourage the authors to put forth hypothetical design experiments that could fill the gaps or consolidate some of the findings in the field, or even tease out the mechanism better. That way this manuscript will have a better impact on the field.
Please break the discussion down to sections of methodological considerations and mechanistic considerations for better readability.

**Reviewer**	**2**
**Name**	**Katie Scandrett**
**Affiliation**	**University of Birmingham**
**Date**	**07-Sep-2023**
**COI**	**07-Feb-2025**

Thank you for the opportunity to review this interesting and well-written manuscript. It seems that the main purpose of the review is not to evaluate the effectiveness of the FMV interventions, but instead to describe various FMVs that have been used in patients with SCIs, since no outcome data has been reported. Given the very low yield of the search (n=90), it cannot be claimed that the literature has been systematically searched. Therefore, I would encourage the authors to re-frame this work as a literature review. My comments below are related to the presentation of the manuscript as a systematic review, although some would also be relevant if the authors decided to re-frame the design of this review.

The first paragraph of the introduction is very long and quite hard to read as a result.

Why was Embase not searched? It is recommended that both MEDLINE/PubMed and Embase are searched when conducting a systematic review.

The inclusion criteria lacks detail. First, the study designs eligible for inclusion are not mentioned at all. More detail is needed to describe each of the PICO elements. For example,

were participants of any age eligible for inclusion? Were there any restrictions based on the timing/frequency of FMVs and the follow-up length? The mentioned outcomes are extremely broad. For example, strength could be assessed in a multitude of ways. Were there any restrictions based on the measurement of the mentioned outcomes? Were studies required to report both or one of these outcomes to be included?

Given the search is over a year out of date, the review authors should consider updating it.

Were title and abstract and full-text screening conducted independently and in duplicate?

Data extraction and quality assessment should be conducted independently and in duplicate, or there should be a strategy for checking agreement between reviewers.

Which effect measures were extracted or calculated from each included study?

Even though no meta-analysis was performed, there should be a description of how results were narratively synthesised.

The domains that the risk of bias tool assesses have not been mentioned. Also, there is no mention of how high, unclear or low risk of bias was determined.

The order that the methods section is reported in isn't logical.

The reasons for exclusion should be given in the PRISMA diagram at the full text screening stage. Were theses not excluded at the title and abstract or full text stage?

The results section mentions the outcomes that seem to have been reported in the included studies. However, no outcome data is reported. Even if no meta-analysis has been conducted, results can still be tabulated and synthesised narratively.

Why were non-disabled individuals included if the population of the review was SCI? Were results only in the SCI individuals considered? If studies where results were presented separately for those with SCI were eligible for inclusion, this should have been mentioned in the inclusion criteria.

Why is the medication that individual patients are taking relevant?

No interpretation of the risk of bias assessment is given.

The results section is categorised based on whether the FMV is used for the upper or lower limb. Any categorisations for narrative synthesis should be described and justified in the

methods section.

The PEDro scale is for assessing the quality of randomised controlled trials (for example, the PEDro scale assesses random allocation and allocation concealment). However, none of the included studies are randomised controlled trials.

A handful of studies that provide mechanistic evidence are mentioned in the discussion, but only one reference is included.

Some interpretation of results that seem to describe the effectiveness of FMV are alluded to in the discussion (i.e. 'The use of FMV in ULs of SCIs present a mixed picture of its potential benefits on mobility'). However, mention of this comes out of nowhere since no results are presented in the results section. These results should be presented fully and coherently, with reference to the comparator intervention in the results section if the aim of the review is to evaluate the effectiveness of FMV.

The discussion is hard to read due to the very long paragraphs.

The conclusion should be placed after the study strengths (which are missing) and limitations section and reflect on findings in light of the identified limitations.

VERSION 1 - AUTHOR RESPONSE

Reviewer: 1
Dr. Santosh Chandrasekaran , Northwell Health
Comments to the Author:
This manuscript attempts to present a systematic review on the effects of Focal Muscle Vibrations (FMVs) on sensorimotor function, and functional gains such as mobility and strength in people with SCI. They included 23 studies, 9 focusing on the upper-limb and 14 on lower-limb interventions. The studies included 422 individuals with SCI and 132 able-bodied individuals.

I have significant concerns that this review does not add to the body of the knowledge. I believe a systematic review should aim to further the field by suggesting future experiments by highlighting the gaps in the existing body of knowledge.

Thanks for your insightful comments and suggestions, we have addressed your concerns point by point, below. Specifically, the manuscript now summarises study strengths and limitations, succinctly after abstract (as suggested by the research editor) and in detail in a new section 6 *Study Strengths and limitations*. The section also makes *Future recommendations*, thus adding to the body of knowledge.

My detailed comments are as follows:

Abstract

Comment 1: Line 35 - "The pathophysiology of SCI...less effective than desired." - This is too vague. For better readability, it should perhaps focus on what is missing and how FMV might fill that gap. Please also briefly include what FMV is and how it is administered.

Reply 1: We have addressed the above comments in the newly formatted and revised abstract. Please see lines 20-25.

Comment 2: "... FMVs, through afferent nerve stimulation..." - This statement is too vague and too strong. Please clarify.

Reply 2: The above elements are now clarified in the newly formatted and revised abstract. Please see lines 20-25.

Comment 2a: Conclusion - "Our findings suggest potential benefits..." - On what percentage of studies and in what context is this statement based? This is again too broad and too strong a statement. Please include in the abstract, briefly, appropriate caveats and percentage of studies that support this conclusion.

Reply 2a: The above elements are now clarified in the newly formatted and revised abstract. Please see lines 40-45.

Intro

Comment 3: Pg 3 - This section of the Intro is too broad and describing the statistics in a lot of detail. In my opinion, it is too distracting, and I would encourage the authors to make it more concise for better readability. Please restructure in a way that it flows for the introduction of FMV in the next section.

Reply 3: The first paragraph is now shortened, and the statistics are presented in a summarised form to make the message more concise and maintain flow. Please see highlighted text throughout the introduction.

Comment 4: Line 40 - "and, pressure ulcers"

Reply 4: This has been now amended. Please see line 64.

Comment 5: Line 41 - complications negatively affect patient's life expectancy

Reply 5: This has been now amended. Please see line 65.

Comment 6: Pg 4, line 6 - "inherent potential of neuromuscular system for promoting functional recovery after neurological injury (such as SCI) or neurological disorders" This is extremely unclear and vague. Please clarify. What do the authors mean by "The neuromuscular system having an inherent potential to promote recovery"? Please introduce and explain this in multiple sentences.

Reply 6: A new description has been added in the second paragraph of introduction section to address this comment. Please see lines 77-88.

Methods

Results

Comment 7: Stage and LC needs to be rephrased for better readability

Reply 7: The word 'stage' is now replaced by 'phase' to represent if SCI is chronic or acute. And the abbreviation 'LC' (level of completeness) is replaced by 'severity' to represent if the injury is complete or incomplete.

Discussion

Section 4.1

Comment 8: Please have separate headings/sections for UL and LL

Reply 8: Both sections 4.1 and 4.2 now have sub-headings for better readability.

Comment 9: Line 27: "mixed picture of its potential benefits..". This language conflicts with the one in the Abstract - "Our findings suggest potential benefits...". Please edit.

Reply 9: Abstract has been rewritten to resolve this conflict.

Comment 10: Line 37: mention level of completeness of SCI

Reply 10: level of completeness of SCI has been clarified throughout the manuscript and the changes are now highlighted.

Comment 11: Line 41: context-dependent. Please be explicit

Reply 11: The explanation has been added for clarification, please see line 396

Comment 12: Line 49: C7 or higher - how high? That might explain why triceps did not show improvement

Reply 12: The sentence now has the range of level of SCI. (It was C4 to C7). Not only this but entire manuscript also now has the information of the level also written while being discussed. Please see line 406.

Comment 13: Pg 14, Line 4 onwards: The authors keep emphasizing that SCI context matters to evaluate FMV effectiveness but seldom mention the context when describing the observed results. I would encourage the authors to mention level of SCI and completeness

Reply 13: We have now added the information about the level and completeness of the SCI in the text as well for all the reported results throughout this review.

Comment 14: Line 17: "brief 5.5 minutes" how is it brief when there's a mention of a 54s FMV intervention

Reply 14: We have removed the word 'brief' to remove the confusion.

Section 4.2
Comment 15: Pg 14, line 36: TVR abbreviation

Reply15: Thank you for pointing this out. The declaration of this abbreviation is now removed from the discussion section, as the first declaration of the abbreviation is already in the Result section 3.3.

Comment 16: Line 44: how was corticomotor excitability evaluated?

Reply 16: A sentence explaining the assessment mode is now added in the section 4.2.1. It was assessed with TMS and motor evoked potentials from the thenar muscle. Please refer to lines 455-456.

Comment 17: Line 60: Please mention what is the clinical effect of Diazepam to provide context - GABA agonist

Reply 17: Diazepam acts on GABA-A and is used in SCI to treat spasms and spasticity. We have now mentioned this. Please see line 424.

Comment 18: Line 60: Please define H/M ratio

Reply 18: Definitions are now added in the text. In section 4.2.2. Please see lines 463-473.

Comment 19: Please define T-reflex
Reply 19: Definitions are now added in the text. In section 4.2.2. Please see lines 497-498.

Comment 20: The authors went to great lengths to list out all the variables encountered in the studies they review - outcome measures, vibrational frequency, vibration amplitudes, duration of FMV administration, number of sessions, relation between target muscle and injury level. However, they make no recommendations for future studies based on these observations. I would encourage the authors to put forth hypothetical design experiments that could fill the gaps or consolidate some of the findings in the field or even tease out the mechanism better. That way this manuscript will have a better impact on the field.

Please break the discussion down to sections of methodological considerations and mechanistic considerations for better readability.

Reply 20: Thank you for the insightful feedback. In response to your recommendation, we have now revised the manuscript to include a 'Future recommendations' section which details the limitations and gaps in the findings and proposes future research designs to address these gaps.

Reviewer: 2
Miss Katie Scandrett, University of Birmingham
Comments to the Author:

Comment 1: Thank you for the opportunity to review this interesting and well-written manuscript. It seems that the main purpose of the review is not to evaluate the effectiveness of the FMV interventions, but instead to describe various FMVs that have been used in patients with SCIs, since no outcome data has been reported. Given the very low yield of the search (n=90), it cannot be claimed that the literature has been systematically searched. Therefore, I would encourage the authors to re-frame this work as a literature review. My comments below are related to the presentation of the manuscript as a systematic review, although some would also be relevant if the authors decided to re-frame the design of this review.

Reply 1: We acknowledge the reviewer concerns on the yield of the search, but this apparent shortfall is not a limitation of the methodology design but rather the dearth of available data in literature for the topic. A previous systematic review [5] on this topic, for example, also confirms this limitation with a similar low yield. And systematic reviews with low yields are not uncommon where new technological intervention is being reviewed.

[5] Sadeghi, M., & Sawatzky, B. (2014). Effects of vibration on spasticity in individuals with Spinal Cord Injury. American Journal of Physical Medicine & amp; Rehabilitation, 93(11), 995–1007. doi:10.1097/phm.0000000000000098

Comment 2: The first paragraph of the introduction is very long and quite hard to read as a result.

Reply 2: This was also recommended by the first reviewer. We have revised the first paragraph made it more concise and readable.

Comment 3: Why was Embase not searched? It is recommended that both MEDLINE/PubMed and Embase are searched when conducting a systematic review.

Reply 3: We do not have Embase subscription.

Comment 4: The inclusion criteria lack detail. First, the study designs eligible for inclusion are not mentioned at all. More detail is needed to describe each of the PICO elements. For example, were participants of any age eligible for inclusion? Were there any restrictions based on the timing/frequency of FMVs and the follow-up length? The mentioned outcomes are extremely broad. For example, strength could be assessed in a multitude of ways. Were there any restrictions based on the measurement of the mentioned outcomes? Were studies required to report both or one of these outcomes to be included?

Reply 4: We have considerably changed the whole methodological section based on the valuable comments of the reviewer. So, it has a greater detail and clarity.

Section 2.1 now has a more detailed description of the PICO criteria. We have now added a few sentences in section 2.2 to further clarify the inclusion criteria. We did not put any restrictions on the age limit of SCI because the injury is not age dependent and can unfortunately happen at any stage of life. Putting restrictions on the FMV parameters was also not an objective since we want to find the effect of different experimental variables on different outcome measures. As it is important for the understanding of the effect of FMV on the neuromuscular performance. For the outcome measures, we have now added a clarification in section 2.2 that only one of

the two conditions need to be satisfied. Both criteria are important because FMV is a considerably new technology and understanding 1) the mechanisms of its action and 2) the different methods for performance enhancement would add intellectual value for future management and possibly treatment of SCIs. We have added a sentence in the last paragraph of 'Introduction' section to highlight this importance. (Reviewer 1 also pointed out that these would have an impact on the field). Since the area is not very populated with research, the outcome measures had to be kept broad to picture the landscape properly.

Comment 5: Given the search is over a year out of date, the review authors should consider updating it.
Reply 5: The review has now been updated to include articles until 15th February 2025. This adds two more articles into the review. Important to add that the search was significantly out of date due to journal taking significantly long time (~8 months) to find reviewers.

Comment 6: Were title and abstract and full-text screening conducted independently and in duplicate?
Reply 6: These were assessed by first and second author, and conflicts were mutually resolved. The text now details this process. Section 2.5.

Comment 7: Data extraction and quality assessment should be conducted independently and in duplicate, or there should be a strategy for checking agreement between reviewers.
Reply 7: The data extraction was performed by reviewer 1 and these were reassessed by reviewer 2. Mutually agreed results were reported. Text edited. Section 2.6.

Comment 8: Which effect measures were extracted or calculated from each included study? Even though no meta-analysis was performed, there should be a description of how results were narratively synthesised.

Reply 8: A description of the result synthesis is now added into the methodology section. The results were synthesized based on the outcome measures, limb of intervention and disease characteristics. Please see lines 166-175.

Comment 9: The domains that the risk of bias tool assesses have not been mentioned. Also, there is no mention of how high, unclear or low risk of bias was determined.

Reply 9: The section 2.7 is now re-written to include the definition of the risk of bias tool and the overall risk calculation method. (The tool has also been changed as a response to a later comment.)

Comment 10: The order that the methods section is reported in isn't logical.
Reply 10: We have re-organised the method section which now contains more sub-sections. The selection of this new heading structure has been based on the structure in this article the Editor suggested above

https://bmjopen.bmj.com/content/12/2/e054120

Comment 11: The reasons for exclusion should be given in the PRISMA diagram at the full text screening stage. Were theses not excluded at the title and abstract or full text stage?
Reply 11: We have updated the PRISMA diagram with the reasons provided in the screening stage. Please refer to Figure 1. Theses were excluded after being retrieved, we have adjusted this into the new figure.

Comment 12: The results section mentions the outcomes that seem to have been reported in the included studies. However, no outcome data is reported. Even if no meta-analysis has been conducted, results can still be tabulated and synthesised narratively.
Reply 12: Outcome data is already reported, please refer to outcome and measures column in Table 2.

Results have been narratively synthesized. Table 2 already provides a detail of the extracted data from the studies, including the outcome measure (sensorimotor function of mobility and strength), disease characteristics, intervention parameters and muscle of action. We have now also added a new column into Table 2 which details the specific outcome measure used for each of the study.

Comment 13: Why were non-disabled individuals included if the population of the review was SCI? Were results only in the SCI individuals considered? If studies where results were presented separately for those with SCI were eligible for inclusion, this should have been mentioned in the inclusion criteria.

Reply 13: Thank you for pointing this out. We have removed the information about non-disabled individuals. This was only presented as a secondary information, but as this does not reflect the main aim of the review, we have now omitted this.

Comment 14: Why is the medication that individual patients are taking relevant?
Reply 14: Effects of vibration might be co-modulated by the medication (i.e. by

diazepam baclofen etc.), hence mentioning this is relevant. Also, some of the studies were of diazepam + vibration. They are relevant studies because they met other criteria, and we should not leave them out because there was a diazepam/medication arm to them.

Comment 15: No interpretation of the risk of bias assessment is given.

Reply 15: The review now has a new section 3.1 that provides insights into the risks of bias in the included studies.

Comment 16: The results section is categorised based on whether the FMV is used for the upper or lower limb. Any categorisations for narrative synthesis should be described and justified in the methods section.

Reply 16: A description and a justification are now added in the 'Methods' section in the sub-section 'Data Extraction'. Section 2.6.

Comment 17: The PEDro scale is for assessing the quality of randomised controlled trials (for example, the PEDro scale assesses random allocation and allocation concealment). However, none of the included studies are randomised controlled trials.

Reply 17: Thanks for pointing this out. We have changed the risk assessment tool. We are now using Risk Of Bias In Non-randomised Studies – of Interventions (ROBINS-I) tool, which is specifically designed to address nonrandomised control trials. Detail of the tool is provided in the methods section.

Comment 18: A handful of studies that provide mechanistic evidence are mentioned in the discussion, but only one reference is included.

Reply 18: Thanks for spotting this, we have now included references to other studies which provide mechanistic evidence.

Comment 19: Some interpretation of results that seem to describe the effectiveness of FMV are alluded to in the discussion (i.e. 'The use of FMV in ULs of SCIs present a mixed picture of its potential benefits on mobility'). However, mention of this comes out of nowhere since no results are presented in the results section. These results should be presented fully and coherently, with reference to the comparator intervention in the results section if the aim of the review is to evaluate the effectiveness of FMV.

Reply 19: Perhaps be the sentence "The use of FMV in ULs of SCIs present a mixed picture.." starts on an incorrect note, we have reworded this statement.

As far as your comment, “these results should be presented fully and coherently, with reference to the comparator intervention”, we believe that in practice with systematic reviews on technological intervention, we have presented all the quantitative elements of the results in detail, fully and coherently (including details about SCI level, outcomes used, physiological analyses/measures, FMV frequencies, amplitudes, durations, locations) with comparator intervention, in result section.

Discussive elements, i.e. interpretations to be drawn from these results which “evaluate the effectiveness of FMV” in detail, fully and in coherent manner, are presented in the discussion section. We believe, this is more about the preference where this information is presented in the review (result or discussion section), rather than this information being absent.

Comment 20: The discussion is hard to read due to the very long paragraphs.

Reply 20: To improve readability, the discussion section now has sub-headings for the upper and lower limb. Also, we have now divided the text into more paragraphs to enhance readability and separate the aspects discussed.

Comment 21: The conclusion should be placed after the study strengths (which are missing) and limitations section and reflect on findings in light of the identified limitations.

Comment 21: The conclusion is now at the end, following the study strength and limitations section. Section 6 is renamed as ‘Study strength and limitations’ and now contains the strengths of the review as well.
